# Sudden sensorineural hearing loss associated with inner ear lesions detected by magnetic resonance imaging

**DOI:** 10.1371/journal.pone.0186038

**Published:** 2017-10-04

**Authors:** Jiwon Cho, Hanjae Cheon, Jung Hye Park, Hyo-Jeong Lee, Hyung-Jong Kim, Hyo Geun Choi, Ja-Won Koo, Sung Kwang Hong

**Affiliations:** 1 Department of Otorhinolaryngology-Head and Neck Surgery, Hallym University Sacred Heart Hospital, Anyang, Republic of Korea; 2 Laboratory of Brain & Cognitive Sciences for Convergence Medicine, Hallym University College of Medicine, Anyang, Republic of Korea; 3 Department of Otorhinolaryngology-Head and Neck Surgery, Seoul National University Bundang Hospital, Seongnam, Republic of Korea; University of Palermo, ITALY

## Abstract

Although recent advances in magnetic resonance imaging (MRI) techniques have contributed to the detection of tiny lesions in the internal auditory canal (IAC) that may be responsible for sudden sensorineural hearing loss (SSNHL), there have been relatively few studies on the clinical characteristics of intra-labyrinthine hemorrhage (ILH) and labyrinthitis versus those regarding IAC tumors. Our purpose was to investigate the frequency of those IAC lesions on MRI and their clinical characteristics. Initial MRIs of 200 patients with SSNHL (93 men, 107 women; mean age = 48.61 years, range: 18–84 years), as well as detailed clinical histories, audiological examinations, and thyroid function, lipid battery, and serological tests (for viral agents and autoimmune disease), were performed. All patients were hospitalized at the time of diagnosis of SSNHL and were administered the same treatment protocol. Patients were divided into idiopathic and secondary groups according to their MRI results. After discharge, they underwent follow-up audiometry and clinical examination at predetermined intervals (2 weeks, 1, 2, 4, and 6 months, and 1 and 2 years). Propensity score-matching and receiver operating characteristics curves of the initial parameters were used for estimating clinical characteristics. Of the 200 patients, 25 (12.55%) who had abnormal findings suggesting inner ear lesions on MRI were assigned to the secondary SSNHL group; within this group, 10 patients (10/200, 5%) had a tumor invading the IAC, 7 (7/200, 3.5%) had ILH, 6 (6/200, 3%) had labyrinthitis, and 2 (1%) had a structural deformity of the IAC. The secondary group showed significantly poor recovery of hearing function compared with that in the idiopathic group. Patients with ILH or labyrinthitis showed prognoses that were equally poor as those of patients with tumors in the secondary group. Additionally, patients with such lesions showed significant canal paresis on the lesion side at an early stage and a high prevalence of benign paroxysmal positional vertigo (BPPV). In conclusion, the prevalence of non-tumorous lesions on MRI represents common findings and showed a poorer treatment response than that of vestibular Schwannoma in patients with SSNHL. Abnormal canal paresis (cut-off value of 35% on the lesioned side, sensitivity 65.2% and specificity 67%), spontaneous nystagmus directed to the contralesional side, and positional vertigo would be the clinical presentation of SSNHL with IAC lesions, in which the presence of acute prolonged vertigo or positional vertigo compatible with BPPV suggests the possibility of a non-tumorous lesion, such as ILH or a labyrinthitis rather than an IAC tumor.

## Introduction

Sudden sensorineural hearing loss (SSNHL) is commonly defined as sudden hearing impairment, of at least 30 dB at three consecutive frequencies occurring over 3 days [1], and its incidence has been estimated as 5–20/10,000 individuals per year [2, 3]. Although the exact etiology of SSNHL is still debated (such that it is classified as idiopathic), viral infections, immunological diseases, and impairment of the vascular microcirculation have been suggested as possible causes [2, 4–6]. Recently, studies have shown that abnormal serum lipid levels [7, 8] and thyroid dysfunction were also significantly associated with the occurrence and prognosis of SSNHL [9]. However, no laboratory test can accurately predict the prognosis of SSNHL and no treatment modality has been established based on medical evidence, despite biological plausibility.

In contrast to laboratory findings, lesions invading inner ear structures, as an identifiable cause, can lead to secondary sudden hearing loss. Approximately 1–6% of patients with SSNHL show a vestibular Schwannoma (VS) on magnetic resonance imaging (MRI), which is known as the most common pathological finding in SSNHL with inner ear lesions [10–12]. Interestingly, meticulous analysis of the inner ear and recent advances in imaging techniques increased the detection rate of non-tumorous isolated labyrinthine lesions, such as labyrinthine hemorrhage and inflammation; the prevalence has been estimated to be 3.8–9%, which revealed a high proportion similar to that of VS [11, 13, 14].

MRI has been criticized as a screening test for the pathological entity of SSNHL due to its low diagnostic yield (high cost per positive test) [15], whereas the auditory brain stem responses (ABRs) have been used widely as a screening procedure for the diagnosis of VS, particularly when MRI is not available. However, this test cannot detect small lesions (<1 cm) [16], and sufficient residual hearing must be present for a recording to be obtained. Furthermore, intra-labyrinthine hemorrhage (ILH), inflammatory lesions, and structural abnormalities are not detectable by ABRs. Thus, MRI could provide valuable information to predict the prognosis and diagnostic process of SSNHL when those lesions were suspected because the clinical characteristics may differ from those of idiopathic SSNHL.

In this article, we focused on the clinical features of patients with inner ear lesions on MRI compared with those with idiopathic SSNHL and further explored the differential clinical characteristics between tumorous and non-tumorous lesions in secondary SSNHL. To probe this, we investigated MRI and the association with the clinical characteristics of 200 patients with SSNHL with a maximum of 879 days follow-up (mean = 510 days).

## Materials and methods

### Ethics

Approval for this study was obtained from the Institutional Review Board of Hallym University Sacred Heart Hospital. All data were collected from our register of patients with SSNHL, in which all data were fully anonymized; thus, informed consent for participant was neither necessary nor possible. Our ethics committee approved this procedure because this work is study involving collection of existing data from the register that subjects could not be identified.

### Subjects

The MRI scans of 280 consecutive patients, diagnosed with SSNHL between May 1, 2006 and November 30, 2016, and their clinical characteristics were analyzed. The audiometric criteria for SSNHL were a rapid decrease in hearing of more than 30 dB affecting at least three consecutive frequencies within 3 days. Subjects who had 1) mixed hearing loss due to middle ear disease, 2) a prior history of sudden deafness or previous ear surgery, and 3) fluctuating hearing loss such as Ménière’s disease were excluded. Additionally, those with MRI scans carried out with a 1.5 Tesla (T) MRI scanner were excluded.

All patients were hospitalized at the time of the diagnosis of SSNHL and were administered the same treatment protocol (daily oral administration of 60 mg prednisolone for 7 days). All MRI scans were obtained within 3 days after admission. Subjects were divided into idiopathic and secondary groups according to the MRI results. They were evaluated by audiometry and clinical examination at predetermined intervals (2 weeks, 1, 2, 4, and 6 months, and 1 and 2 years).

### Initial neuro-otological and laboratory tests

Detailed neuro-otological tests including pure tone audiometry, spontaneous nystagmus (SN), head-shaking nystagmus (HSN), positional nystagmus (PN), a bithermal caloric test and laboratory tests including lipid battery, thyroid function, and serological tests (HBsAg, Anti HBs, Anti HCV, HIV) were performed on the day of admission. Pure-tone thresholds were obtained at frequencies of 250, 500, 1,000, 2,000, 3,000, 4000 and 8000 Hz with calibrated pure-tone audiometry (GSI AudioStar Pro; Grason Stadler, Eden Prairie, MN) in a soundproof audio booth. The pure-tone average (PTA) was calculated as an average of the pure-tone thresholds measured at 500, 1000, 2000, and 3000 Hz, which was used interchangeably with subjects’ hearing level in this article.

The slow-phase velocities of the SN, HSN, and PN were measured using a videonystagmography system (System 2000; Micromedical, Chatham, IL) in the sitting position. SN was recorded over 20 s in the same position with the eyes open. HSN was assessed after 15 s of passive head shaking at a frequency of 2 Hz with 30° neck flexion. Nystagmus was considered significant when the degree was ≥ 3°/s. Vertigo was defined as distorted sensation of self-motion according to International Classification of Vestibular Disorders I [17]. PN was measured during sitting, lying down, supine roll (head turning to the left, head center, and then to the right in a supine position), sitting up, head bending, and the bilateral Dix-Hallpike position, in that order, and the diagnosis of benign paroxysmal positional vertigo (BPPV) was based on clinical practice guidelines developed by the American Academy of Otolaryngology-Head and Neck Surgery Foundation [18]. The bithermal alternating caloric test was performed using an Aquastar water caloric stimulator (Micromedical), in the supine position, with the head elevated by 30°. The results were considered abnormal when the canal paresis (CP) score was ≥ 25%.

### MRI acquisition and diagnostic criteria

All MRI scans were obtained with a 3 T MR unit (Achieva; Philips Healthcare, Best, the Netherlands). The MRI protocol consisted of T1-weighted images (T1WIs), contrast-enhanced (CE)-T1WIs, -T2WIs, -3D volume isotropic turbo spin-echo acquisition (VISTA), and 3D fluid-attenuated inversion recovery (FLAIR)-VISTA. The scan parameters for 3D-FLAIR were: repetition time, 4,800 ms; effective echo time, 274 ms; inversion time, 1,650 ms; flip angle, 90° (constant); turbo spin-echo refocusing echo train length, 140; and matrix size, 256 × 256. Inner ear lesions were reviewed by an experienced neuroradiologist. ILH was considered a high signal on the pre-contrast T1WI sequence and 3D FLAIR VISTA. Labyrinthitis was considered a high signal on CE T1WIs and 3D FLAIR VISTA. Tumorous lesions in the inner ear were identified on CE-T1W1. Subjects with lesions detected by MRI were assigned to the secondary SSNHL group.

### Outcome measures and statistical analysis

Hearing improvement was defined as slight improvement (SI, > 15 dB gain and a final hearing level > 45 dB), a partial recovery (PR, > 15 dB gain, final hearing level 25–45 dB), or complete recovery (CR, final hearing level < 25 dB) according to the last PTA during follow up [19].

Group comparison for various parameters, including age, underlying disease, initial hearing level, vertigo symptoms, accompanied by BPPV, neuro-otologic and laboratory findings, and treatment response between idiopathic and secondary SSNHL identified by MRI, were performed using independent sample t-tests and χ2 tests according to the characteristics of the covariates. For comparison of the treatment response between groups, we used propensity score matching for optimal comparison of prognoses. Based on the calculated propensity score, and using logistic regression including the covariates of gender, age, underlying disease, dyslipidemia, thyroid function, and initial hearing level of patients in the secondary SSNHL group, each patient was matched with one from the idiopathic SSNHL group according to an optimal algorithm (case-control matching according to the propensity score). The log-rank test was used to compare the prognosis of SSNHL between the two groups. In our study, the definition of an event was defined as hearing recovery more than PR, and the analysis was based on the time of the event for the individual follow-up period, obtained from the subject’s statement and follow-up audiometry. If this event did not occur until the last follow-up date or the end time point of this study, the subjects were censored at those time points. Of the 280 patients initially enrolled in the study, 80 (80/280, 28.6%) were lost to follow up at the early time or declined participation due to other causes. Thus, 200 patients with SSNHL (93 men, 107 women; mean age = 48.61 years, range: 18–84 years) were included in the final analysis. The length of the follow up of participants varied, ranging from 4 to 879 days (mean = 510 days). All statistical analyses were two-tailed, and a *p*-value < .05 was considered to indicate statistical significance. Calculations were performed using SPSS for Windows software (ver. 22.0; SPSS Inc., Chicago, IL).

## Results

### Initial comparison data between the idiopathic and secondary groups

**Fig 1:**
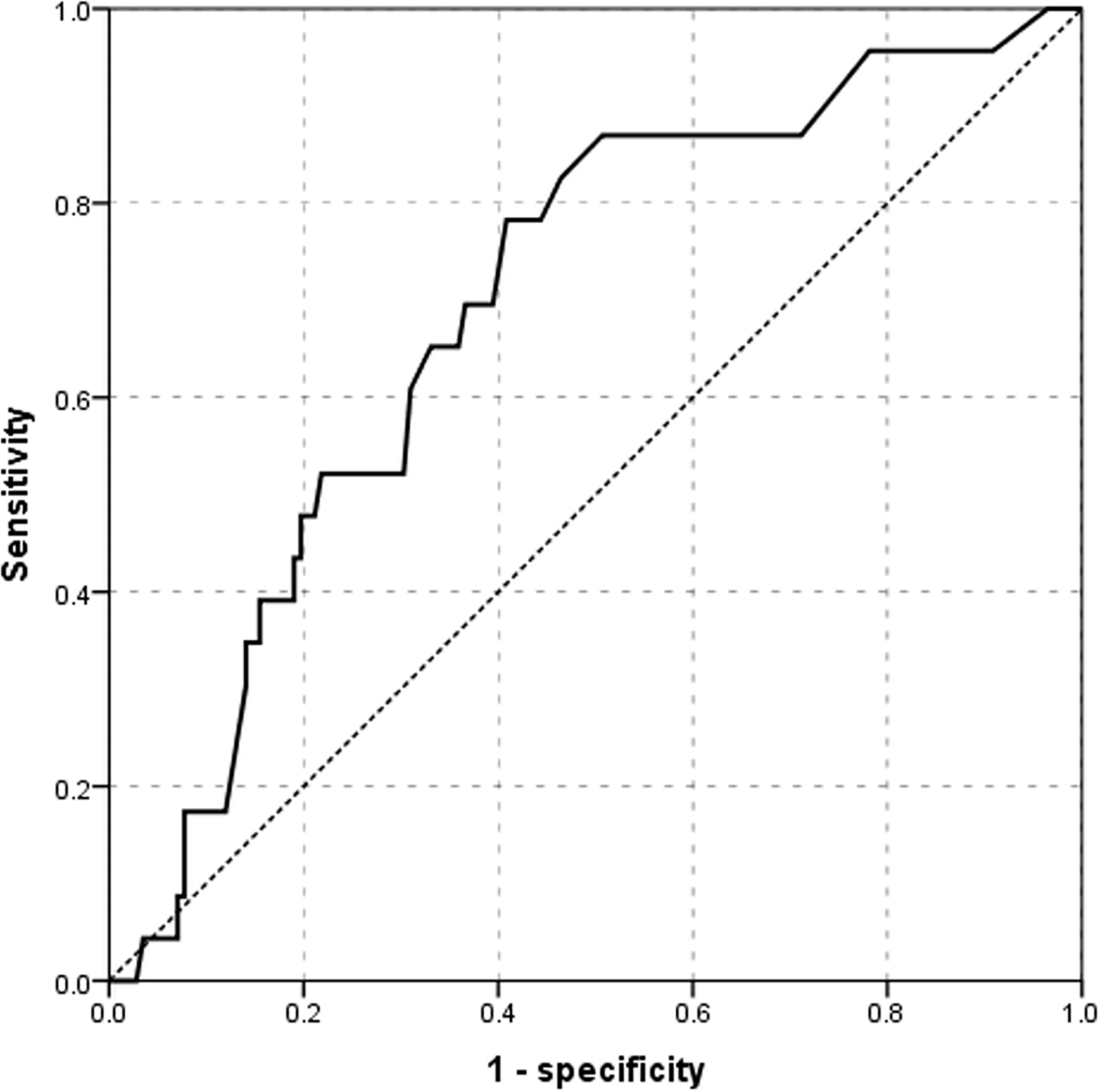
Receiver operating characteristic (ROC) curves of canal paresis (CP) on the lesion side according to magnetic resonance imaging (MRI). The ROC curves of the CP were plotted according to the patient group, as discriminated by MRI (idiopathic vs. secondary). The area under the curve for CP was 0.695, and the cut-off value was 34.5% (sensitivity 65.2%, specificity 67%).

Overall, 200 patients with SSNHL (93 men, 107 women; mean age = 48.61 years, range: 18–84 years) were finally included. Among them, 10 patients (5%) had a tumor invading the internal auditory canal (IAC), 7 (3.5%) had an ILH, 6 (3%) had labyrinthitis, and 2 (1%) had a structural deformity (congenital inner ear abnormality) of the IAC; Thus, 25 (12.5%) patients had abnormal imaging findings suggesting inner ear lesion on MRI; they were assigned to the secondary SSNHL group. The mean ± 2 standard deviations of age was 48.70 ± 16.75 years in the idiopathic group and 48.57 ± 18.94 years in the secondary SSNHL group, and those of PTA were 68.54 ± 27.30 dB in the idiopathic group and 72.28 ± 28.19 in the secondary group at the initial time; there was not a statistically significantly difference between the groups (*p >* .05). Significant SN directed to the contralesional side (paralytic nystagmus) was detected in 25 (25/175, 14.2%) subjects, ranging from 3–23°/s, and significant SN directed to the lesional side (irritative nystagmus) was detected in 11 (11/175, 6.2%) patients, ranging from 3–29°/s in the idiopathic group. Significant SN was observed in nine patients, ranging from 3–17°/s and directed exclusively to the contralesional side in the secondary group. Significant HSN directed to the contralesional and lesion sides was found in 37 (37/175, 21.1%) subjects, ranging from 3–28°/s, and 34 (34/175, 19.4%) subjects, ranging from 3–64°/s, respectively, in the idiopathic group. HSN directed to the contralesional and lesion sides was found in 11 (11/25, 44%) subjects, ranging from 3–34°/s, and 4 (4/25, 16%) subjects, ranging from 3–54°/s, respectively, in the secondary group. Four patients who showed HSN directed to the lesion side did not have significant SN. Abnormal CP (> 25%) on the lesion side was present in 56 (56/175, 32%) subjects, ranging from 27–100%, and was present on the contralesional side in 5 (5/175 2.8%) patients, ranging from 34–98% in the idiopathic group. In the secondary group, abnormal CP was found in 20 (80%, 20/25) patients, exclusively on the lesion side. There were significant differences in abnormal CP prevalence (*p* < .001) and SN direction (*p* = .007) between the two groups (Table 1). Interestingly, the occurrence of BPPV was 20% in the secondary group, a value that that was statistically significantly higher than those of the idiopathic group (*p* = .04). The receiver operating characteristic (ROC) curve for CP on the lesion side was plotted; the area under the ROC curve of CP was 0.695. Applying a CP cut-off value of 34.50, the sensitivity and specificity were 65.2% and 67%, respectively (Fig 1).

**Table 1:** Comparison data between the idiopathic and secondary groups.

	Idiopathic sudden hearing loss group (n = 175)	Secondary sudden hearing loss group (n = 25)	*p*- value
**Age (years)**	48.70 ± 16.75	48.57 ± 18.94	NS
**Initial hearing level (dB)**	68.54 ± 27.30	72.28 ± 28.19	NS
**Number of patients with significant SN (%)**	36 (20.4)	9 (36)	.007
Lesional side	11 (6.2)	0 (0)	
Contralesional side	25 (14.2)	9 (36)	
**Number of patients with significant HSN (%)**	71 (40.6)	15 (60)	NS
Lesional side	34 (19.4)	4 (16)	
Contralesional side	37 (21.1)	11 (44)	
**Number of patients with abnormal CP (%)**	61 (34.8)	20 (72)	< .001
Lesional side	56 (32)	20 (72)	
Contralesional side	5 (2.8)	0 (0)	
**Number of patients with BPPV (%)**	13 (7.4)	5 (20)	.04

Values are mean±2SD

SN: spontaneous nystagmus, HSN: head-shaking nystagmus, CP: canal paresis, BPPV: benign paroxysmal positional vertigo, NS: non-significant.

### Clinical characteristics of the secondary group (non-tumorous vs. tumorous lesion)

**Fig 2:**
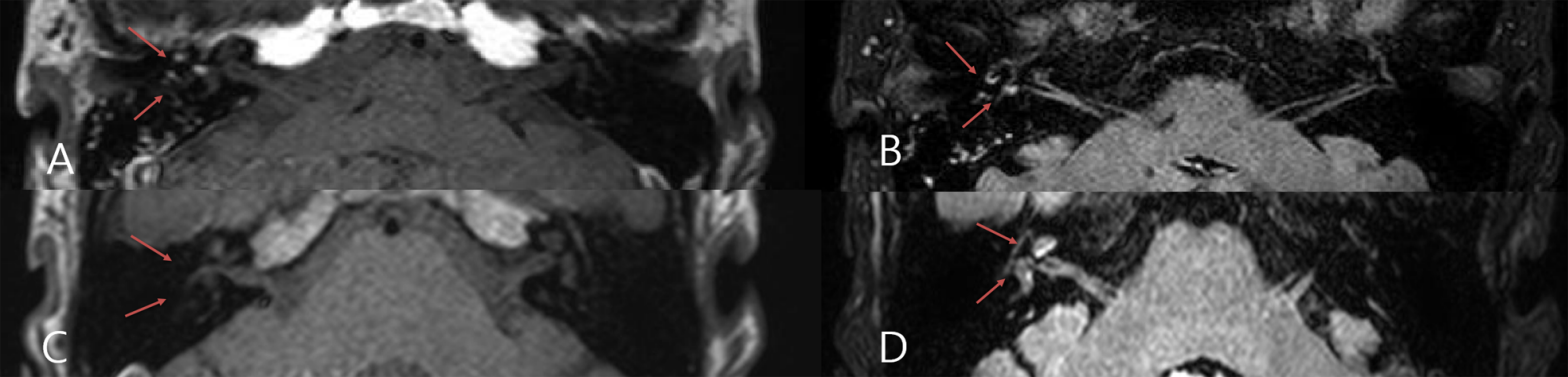
**MRI findings of intra-labyinthine hemorrhage (A,B) and labyrinthis (C,D).** Labyrinthitis and intra-labyrinthine hemorrhage (ILH) both show a high signal intensity on a 3D fluid-attenuated inversion recovery sequence (B,D), but the high signal intensity on the precontrast T1 sequence suggests labyrinthine hemorrhage (A) rather than labyrinthitis (C).

In the secondary group, 10 (10/25, 40%) patients had a tumor invading the IAC (VS: 9 patients; meningioma: 1 patient), 7 (7/25, 28%) had an ILH (Fig 2A–2C)), 6 (6/25, 24%) had labyrinthitis (Fig 2D–2F), and 2 (2/25, 1%) had a structural deformity (congenital inner ear abnormality) of the IAC.

Table 2 lists the demographic characteristics of the subjects with secondary SSNHL (data from two patients with an IAC abnormality not shown here). In comparisons of clinical characteristics by lesion classification, there was no significant difference in age, initial hearing, treatment response, or underlying vascular risk factors between the ILH, VS, and labyrinthitis groups. Accompanying vertigo was present in six of seven patients with ILH, and in three of six patients with labyrinthitis, whereas none of the ten patients with IAC tumors complained of vertigo; these differences between tumorous and non-tumorous lesions were significant (*p* = .001). Additionally, SN to the contralesional side was present in four (4/7) patients with ILH, and in four (4/6) patients with labyrinthitis, whereas only one patient presented with contralesional SN in the VS group; these differences between tumorous and non-tumorous lesions were significant (*p* = .001). HSN directed to the contralesional side and the lesion side were found in five (5/7, 71.4%) and one (1/7, 14.2%) subjects, respectively, in the IHL group, in one (1/10, 10%) and one (1/10, 10%) subject, respectively, in the IAC tumor group, and in four (4/6, 66.6%) and one (1/6, 16.6%) subjects, respectively, in the labyrinthitis group; these differences between tumorous and non-tumorous lesions were significant (*p* = .006). Interestingly, patients with a tumor in the IAC did not correspond with those with BPPV, whereas three (3/7, 42.8%) patients in the ILH group and two (2/6, 33%) in the labyrinthitis group complained of positional vertigo, diagnosed as BPPV; these differences between the tumorous and non-tumorous lesions were significant (*p* = .003). However, there is no significant difference in the abnormal CP and prevalence of underlying vascular disease between tumorous and non-tumorous lesions.

**Table 2:** Demographic findings in patients with inner ear lesions as detected by magnetic resonance imaging.

	Tumor in the internal auditory canal (n = 10)	Intralabyrinthine hemorrhage (n = 7)	Labyrinthitis (n = 6)
**Age (years)**	52.8 ± 19.38	47.57 ± 19.1	36.67 ± 18.61
**Initial hearing level (dB)**	72.1 ± 26.76	72 ± 26.76	75.83 ± 21.07
**Underlying vascular disease (n)**	5	5	3
**Last hearing level (dB)**	60 ± 30.09	82.17 ± 41	66.5 ± 44.28
**Hearing improvement (N)**			
Complete response	1	1	2
Partial response	3	2	0
Slight improvement	0	0	1
**Vertigo (N)** ^**a**^	0	6	3
**Spontaneous nystagmus (N)**^**a**^			
NO	9	3	2
Lesional side	0	0	0
Contralesional side	1	4	4
**Head-shaking nystagmus (N)**^**a**^			
NO	8	1	1
Lesional side	1	1	1
Contralesional side	1	5	4
**Canal paresis (N)**			
NO	3	0	2
Lesional side	7	7	4
Contralesional side	0	0	0
**BPPV (N)**^**b**^			
Posterior semicircular canal	0	1	1
Lateral semicircular canal	0	2^b^	1^b^

The data from two patients with a congenital inner ear abnormality are not shown.

The values are expressed as means ± 2SD.

^a^Item with statistical significance in the group comparison of tumorous and non-tumorous lesions

^b^Cupulolithiasis.

### Follow-up results after propensity score matching

The duration of follow-up was 4–879 days. No difference in treatment response was found between the groups. Considering the various risk factors influencing the prognosis of SSNHL, we used propensity score matching. Specifically, 23 patients from the idiopathic SSNHL group and 23 from the secondary SSNHL group after excluding two patients with IAC abnormality were paired according to propensity scores. Covariates, including underlying disease, lipid levels, thyroid function, initial hearing level, and neuro-otologic parameters, were similar between the groups after propensity score matching, except for the last audiometric threshold. The initial hearing level (PTA) on the lesioned side was 65.87 ± 22.70 dB in the idiopathic group and 74.83 ± 27.96 dB in the secondary group. Overall, CR was achieved in 16 (34.7%, 16/46) patients during follow-up. Although there was no significant difference in initial hearing level between the groups, the last hearing threshold and number of patients with CR was 33.70 ± 24.12 dB and 12 (12/23, 52%) in the idiopathic group and 67.91 ± 36.51 dB and 4 (4/23,17.3%) in the secondary group, respectively, showing a statistically significantly poorer response in secondary SSNHL (Table 3).

**Table 3:** Hearing recovery between the two groups after propensity matching.

	Idiopathic group (n = 23)	Secondary group (n = 23)	*p*-value
**Age (years)**	46.91 ± 14.14	48.65 ± 19.38	NS
**Initial hearing level (dB)**	65.87 ± 22.70	74.83 ± 27.96	NS
**Last hearing level (dB)**	33.70 ± 24.12	67.91 ± 36.51	.001
**Hearing improvement (N)**			
**Complete response**	12	4	.014
**Complete/partial response**	15	9	NS (.07)
**Complete/partial response /slight improvement**	19	10	.007

The values are expressed as means±2SD.

NS: non-significant.

**Fig 3:**
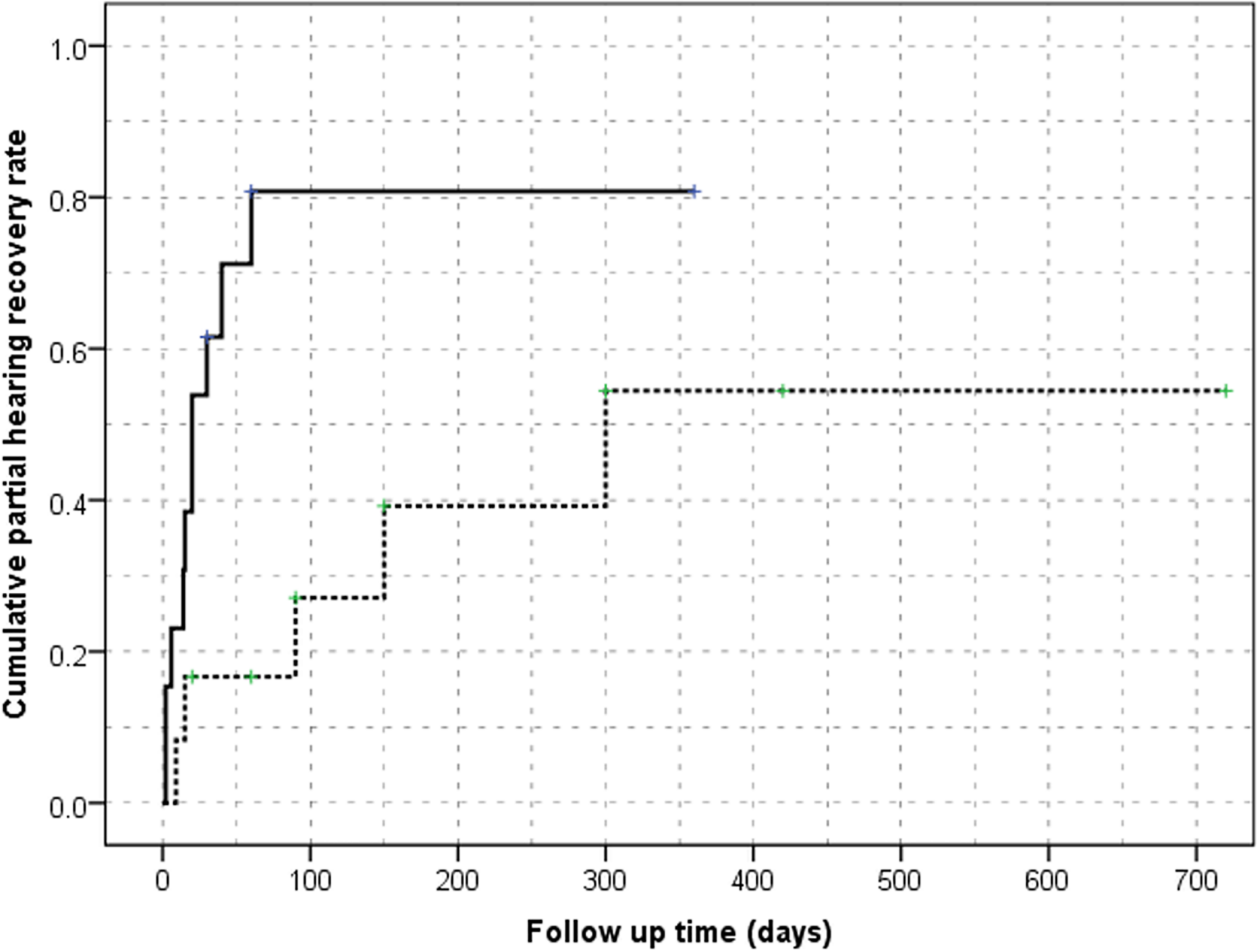
Cumulative partial response rates of patients with normal MRI findings (broken line) and those with an ILH or labyrinthitis (solid line) after propensity score matching. The hazard ratio of a poor response for patients with ILH or labyrinthitis was 1.606 (95% confidence interval: 1.03–2.50).

We focused more on the prognosis in 13 patients with ILH or labyrinthitis (Table 4) because VS or inner ear abnormality may result in irreversible hearing deterioration. The log-rank test revealed that patients with ILH and labyrinthitis also had little chance of recovery during follow-up compared with those in the idiopathic group after excluding patients with VS and inner ear abnormality (*p =* .03; Fig 3).

**Table 4:** Clinical characteristics and follow-up hearing level of 13 patients with non-tumorous lesions.

	Age	sex	Vertigo	Initial hearing (dB)	Last hearing (dB)	SN (deg/sec)	HSN (deg/sec)	CP (%)	Vascular risk factor	History of Anticoagulant^a^	BPPV	Thyroid function	Diagnosis by MRI
1	19	M	+	98	120	15	17	64	-	-	LSC (cupulo)	-	ILH
2	25	F	+	62	6	3	6	60	-	-	PSC	-	ILH
3	43	M	+	101	71	6	10	31	+	-	-	-	ILH
4	54	M	+	28	94	17	33	67	+	+	LSC (cupulo)	-	ILH
5	63	F	+	24	20	0	0	27	+	-	-	-	ILH
6	63	F	-	120	91	2	5	35	+	-	-	-	ILH
7	66	M	+	101	111	0	-11^b^	35	+	+	-	+	ILH
8	19	F	-	102	116	8	18	7	-	-	-	-	Labyrinthitis
9	21	M	-	64	120	0	-54^b^	2	-	-	LSC (cupulo)	-	Labyrinthitis
10	22	F	+	62	21	5	18	70	-	-	-	-	Labyrinthitis
11	49	M	-	61	20	9	34	63	+	-	-	-	Labyrinthitis
12	52	M	+	62	51	8	9	92	+	-	-	-	Labyrinthitis
13	59	F	+	104	71	0	0	87	+	-	PSC	-	Labyrinthitis

SN: spontaneous nystagmus, HSN: head-shaking nystagmus, CP: canal paresis, BPPV: benign paroxysmal vertigo, ILH: intralabyrinthine hemorrhage, MRI: magnetic resonance imaging, PSC: posterior semicircular canal, LSC: lateral semicircular canal.

^a^History of anticoagulants does not include aspirin.

^b^ Direction to lesional

## Discussion

Idiopathic SSNHL is defined as SSNHL with no identifiable cause despite an adequate investigation, up to 90% of which is presumably attributable to vascular, viral, and multiple etiological factors that usually do not require urgent intervention beyond the administration of corticosteroid as initial therapy for a good prognosis [20]. However, because VS, stroke, and malignancies can give rise to SSNHL, as obvious potential causes they should be addressed in the initial and follow-up management course in all patients with SSNHL. In particular, the high prevalence of retro-cochlear lesions, such as VS, in sensory hearing loss (SHL) suggests that all patients with SSNHL should be evaluated for VS [20].

MRI of the IAC has been considered the gold standard for the detection of VS and is more cost-effective than ABR [21]. Additionally, recent advances in MRI techniques (e.g., gadolinium-enhanced or constructive interference in steady state MR sequences) allow accurate assessment of other pathological processes, such as non-tumorous isolated labyrinthine lesions in SSNHL. For instance, a high signal intensity of the inner ear usually on CE-T1WIs and enhanced FLAIR imaging suggests labyrinthitis or hemorrhage, but the high signal intensity of labyrinthitis is substantially decreased on pre-contrast T1WIs.

ILH may cause sudden hearing deterioration with vertigo in a patient on anticoagulant therapy or with a hematological disease; the prevalence has been reported as 0.3–6.25% in patients with SSNHL [7, 22, 23]. In our series of 200 3-T MRI scans, seven (3.6%) patients showed ILH on MRI, which was the second-most common finding after tumors invading the IAC (10 patients, 5.2%). Diffuse abnormal enhancement suggesting labyrinthitis was observed in six (3.1%) patients on post-contrast T1W images and, given that labyrinthitis may be preceded by ILH, in patients with SSNHL the possibility of ILH or labyrinthitis should also be considered to the same extent as VS. Of course, a diagnosis of ILH should also be based on the detailed clinical history, but only two patients had a history of previous anti-coagulation medications, indicating that ILH may also be idiopathic.

The age of the subjects with an IAC lesion detected by MRI was 48.57 ± 18.94 years, which was not significantly different from that of the idiopathic group (48.70 ± 16.75 years). Initial PTA also did not show significant difference between the groups (*p* > .05), inconsistent with earlier studies [14, 23, 24]. SN was frequently present, in four (4/7, 57%) patients with ILH and four (4/6, 66%) patients with labyrinthitis, and the direction was exclusively to the contralesional side (paralytic nystagmus). However, only one (1/10, 10%) patient showed SN in the VS group, indicating that change in static balance depends on the progression of IAC lesions (slow vs. rapid progression). Moreover, three (3/7, 42%) patients with ILH and two (2/6, 33%) with labyrinthitis showed accompanying positional vertigo, diagnosed as BPPV, indicating a significantly higher prevalence than in the idiopathic (13/175, 7.4%) and VS (0/10, 0%) groups (Tables 1 and 2). These findings were not surprising because inflammation of the inner ear or hemorrhagic reaction may injure the anatomical structure of the vestibular labyrinthine, which may in turn result in detachment of otoconia.

Abnormal CP (≥ 25%) on lesion side was present in 56 (56/175, 32%) subjects, ranging from 27–100%, and abnormal CP on the contralesional side was present in 5 (5/175 2.8%) patients, ranging from 34–98%, in the idiopathic group. In the secondary group, abnormal CP was found in 20 patients (80%, 20/25), and the direction was exclusively on the lesion side. Thus, there were significant differences in the prevalence of abnormal CP between the two groups (*p* < .001), so that a CP value > 34.5% suggests the possibility of an inner ear lesion in SSNHL (sensitivity 65.2%, specificity 67%) by ROC curve analysis. If SN to the contralesional side or BPPV accompanies a CP > 34.5%, MRI should be performed for the detection of IAC lesions. However, our findings should be substantiated with further evidence-based studies.

When we focused on clinical presentation in the secondary SSNHL group, there was no significant difference in age, initial hearing, treatment response, or underlying vascular risk factors between the tumorous and non-tumorous lesions. Accompanying vertigo was present in six of seven patients with ILH and three of six patients with labyrinthitis, while none of the ten patients with IAC tumors complained of vertigo; these differences were significant (*p* = .001). SN to the contralesional side was present in four (4/7) patients with ILH, and in four (4/6) patients with labyrinthitis, whereas only one patient presented with contralesional SN in the VS group; these differences were significant (*p* = .001). Taken together, significant SN, abnormal CP, vertigo, and BPPV may be indicators for IAC lesions, and, more specifically, additional acute vertigo accompanied with significant SN directed to contralesional side or BPPV would be a clinical presentation suggesting non-tumorous lesions on IAC rather than tumorous lesions on IAC.

The duration of follow-up was 4–879 days. No difference in treatment response was found between the idiopathic and secondary groups. However, considering the various risk factors influencing the prognosis of SSNHL, we used propensity score matching. In fact, 23 patients in the idiopathic SSNHL group and 23 in the secondary SSNHL group were paired according to their propensity scores. Their initial hearing level of the lesioned group was 65.87 ± 22.70 dB in the idiopathic group and 74.83 ± 27.96 dB in the secondary group after matching, showing the same initial hearing level in the two groups (*p* > .05).

Overall, CR was achieved in 16 (34.7%, 16/46) patients during follow-up. Although there was no significant difference in the initial PTA between the groups, the last PTA and number of CRs was 33.70 ± 24.11 dB and 12 (12/23, 52%) subjects, respectively, in the idiopathic group versus 67.91 ± 36.51 dB and 4 (4/23, 17.3%) subjects in the secondary group. These differences were statistically significant with respect to the prognosis of SSNHL (*p* < .05; Table 3), which is intuitive because VS or inner ear abnormality usually results in irreversible hearing deterioration due to direct invasion. However, log-rank analysis showed that patients with ILH and labyrinthitis had little chance of recovery during follow-up compared with those in the idiopathic group after excluding those patients (*p =* .03; Fig 3). Interestingly, one patient with VS achieved CR (initial PTA 71 dB, last PTA 19 dB), suggesting that hearing recovery does not necessarily indicate that neoplasms are absent in SSNHL, as reported previously [25]. Thus, evaluation of patients with SSNHL for IAC tumors cannot be omitted, even in patients with complete hearing recovery.

## Conclusion

We sought to validate the clinical characteristics of isolated lesions as ILH or labyrinthitis versus tumorous lesions on IAC. The secondary SSNHL group was associated with a poor prognosis, in which ILH and labyrinthitis were the most common findings (6.5%), and their clinical characteristics also differed from those of the VS group as well as those from the idiopathic SSNHL group. Although our results should be confirmed in larger scale studies due to the small number of IAC lesions and low diagnostic yield of abnormal CP (>35%) on caloric tests (sensitivity: 65.2%; specificity: 67%), patients with severe abnormal CP and with SN directed to the contralesional side and BPPV, should be examined by MRI in addition to detailed clinical testing. Clinicians should also consider the possibility of a non-tumorous inner ear lesion, especially in patients with vascular risk factors, acute vertigo with abnormal CP, and BPPV.

## Supporting information

S1 File Raw data from our register. (XLSX)
